# THEORETICAL FRAMEWORK FOR AN ARTIFICIAL INTELLIGENCE–BASED COMPREHENSIVE GERIATRIC ASSESSMENT

**DOI:** 10.1093/geroni/igad104.2823

**Published:** 2023-12-21

**Authors:** Adam Golden

**Affiliations:** University of Central Florida College of Medicine, Orlando, Florida, United States

## Abstract

Comprehensive Geriatric Assessment (CGA) is a process where healthcare professionals evaluate and develop a management strategy for older adults who may have multiple multifactorial illnesses and geriatric syndromes. CGA has traditionally been a labor-intensive process that assesses many factors at a specific point in time. Its availability and feasibility in busy clinical settings is remains limited. Artificial Intelligence (AI) can potentially expand the accessibility and effectiveness of this currently limited resource. By integrating large numbers of longitudinal data sets, AI can evaluate in real time a broad array of ever-changing factors that include medical assessments, diagnostic testing/imaging, laboratory analysis, nursing evaluations, behavioral health interventions, social determinants, electronic monitoring, wearables, community resources, and caregiver support. A geriatric AI framework will need develop computational models that integrate changing biologic, sociodemographic, and mental health factors. The framework could also account for the changing availability of local resources and plan eligibility. The integration of large longitudinal datasets would allow for prediction modeling related to diagnosis, management strategies, and outcomes. Treatment protocols can be automated too. Machine learning will allow for the adjustment of medical alert thresholds to minimize alert fatigue that plaques current electronic health record systems. Just-in-time information can identify appropriate home technology equipment and referrals for community services. An AI framework potentially offers a more comprehensive and longitudinal geriatric evaluation that avoids delays in care while waiting for a single physician to “get to see the patient.”

